# Metachronous pancreatic cancer 18 years after resection of common bile duct cancer: A case report

**DOI:** 10.3389/fsurg.2022.851524

**Published:** 2022-08-24

**Authors:** Seoung Hoon Kim

**Affiliations:** Organ Transplantation Center, National Cancer Center, Goyang-si, South Korea

**Keywords:** pancreatic cancer, common bile duct cancer, metachronous double cancer, survival, follow-up

## Abstract

We report an extremely rare case of metachronous double cancers of the bile duct and pancreas in a single patient who underwent successful curative resections consecutively. At the age of 57, a woman underwent pylorus-preserving pancreaticoduodenectomy for a lesion that was pathologically diagnosed as moderately differentiated adenocarcinoma of the distal common bile duct. Eighteen years later, a pancreatic mass was detected during a follow-up examination. Abdominal computed tomography showed a bigger 2.3 cm lesion at the remnant pancreas body, which suggested a diagnosis of primary pancreatic cancer or metastasis. After admission and further work-up confirming no other lesions, completion total pancreatectomy was performed. The pathological diagnosis of the resected specimen was moderately differentiated pancreatic ductal adenocarcinoma, and this case highlights the occurrence of metachronous double primary cancers developed in both the distal bile duct and the pancreas with an interval of 18 years. This is the first report on the metachronous primary cancers of the bile duct and pancreas with a long interval within an English review of the literature in the MEDLINE. This case serves as another data point to guide surgeons that they should be vigilant for the postoperative long-term surveillance of patients with pancreatobiliary cancer.

## Introduction

Multiple primary cancers are defined by the presence of two or more histologically distinct malignant tumors that are not caused by recurrence, metastasis, or local spread in the same patient and classified as either synchronous tumors or metachronous tumors according to whether the diagnostic intervals of the lesions are shorter or longer than 6 months, respectively (1). The risk of second malignancies has recently increased in cancer survivors because of the development of diagnostic and therapeutic techniques and extension of the human life span (2).

The cancers of the pancreas and biliary tract remain one of the most horrendous oncological diagnoses with an ominous prognosis. With respect to metachronous double pancreatobiliary malignancy, 12 cases of metachronous biliary tract cancer involving gall bladder and bile duct were published in world literature (3). Six cases on completion total pancreatectomy were reported for recurrent pancreatic cancer in the remnant pancreas (4). Also, a case of metachronous cancer of the gallbladder and pancreas was reported in a patient with pancreaticobiliary maljunction, and pancreatic adenosquamous carcinoma was found 5 years after the occurrence of gallbladder adenocarcinoma (5).

However, to date, no case has been reported on a metachronously combined bile duct and on pancreas cancers in the occurrence sequence, even with such a long term separated between them.

Herein, we report the case of a 75-year-old woman who had metachronous pancreatic cancer 18 years after undergoing pylorus-preserving pancreaticoduodenectomy (PPPD) for common bile duct cancer.

## Case presentation

**Figure 1:**
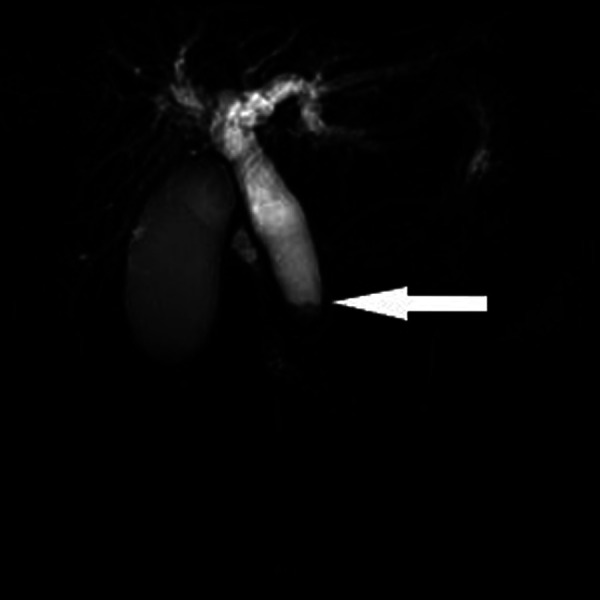
Magnetic resonance imaging showing complete obstruction of the distal bile duct (white arrow).

**Figure 2:**
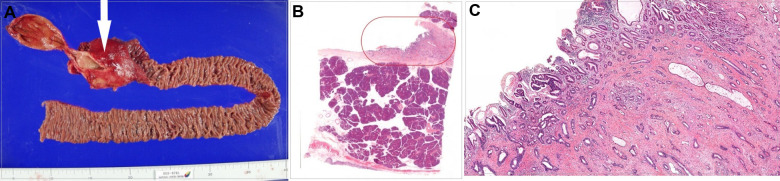
(**A**) Gross specimen of pylorus-preserving pancreaticoduodenectomy, with the white arrow indicating cancer. (**B**) Histologic findings of the distal common bile duct. (**C**) Magnified image of the orange closed curve in (**B**), showing moderately differentiated adenocarcinoma cells of the distal common bile duct.

A 75-year-old woman was admitted for further evaluation of pancreatic mass detected on follow-up CT scan. Eighteen years earlier, she had undergone PPPD for distal bile duct cancer. At that time, her chief complaint was jaundice and the serum level of total bilirubin was 8.4 mg/dl. Magnetic resonance cholangiography showed complete obstruction of the common bile duct (Figure 1). So, percutaneous transhepatic biliary drainage was performed to reduce the jaundice before PPPD. The pathologic specimen showed a 1-cm-sized hard mass of periductal infiltrative type and revealed a moderately differentiated adenocarcinoma with invasion of the pancreas and the ampulla of Vater and with negative resection margins; the tumor stage was T3N0M0, stage IIA, based on the TNM classification of malignant yumors. A histological evaluation of the background pancreas showed normal pancreas (Figure 2).

The woman received adjuvant radiation therapy with concurrent chemotherapy of 5-fluorourocil. Then, she was followed up periodically approximately every 3 months for the first 3 years after surgery, and then approximately every 6 months for the next 6 years, and thereafter once a year without evidence of tumor recurrence. The patient achieved an 18-year recurrence-free survival after the initial resection. However, 18 years later, a follow-up CT scan showed a pancreatic mass. A study of her detailed medical history revealed that there was no family history of cancer as well as no major risk factor for cancer. She had been taking antihypertensive agents for 10 years. She is a nonsmoker and housewife.

**Figure 3:**
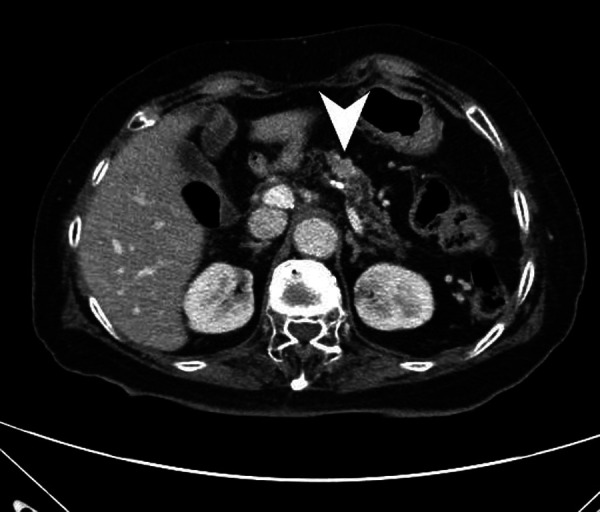
Abdominal CT revealed a big lesion (arrowhead) at the remnant pancreas body.

**Figure 4:**
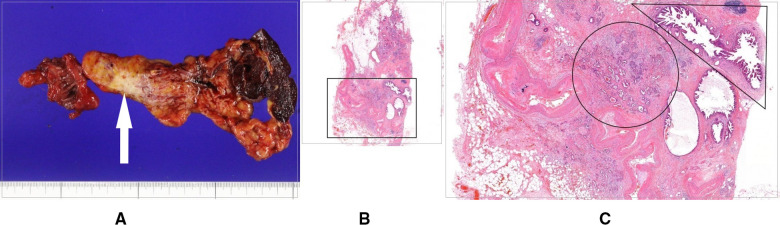
(**A**) Gross specimen of completion total pancreatectomy comprising the remnant pancreas body, spleen, and a jejunal segment including pancreaticojejunostomy, with the white arrow indicating cancer. (**B**) Histologic findings of the pancreas body including cancer. (**C**) Magnified image of the black square in (**B)**, showing pancreatic ductal adenocarcinoma cells (black circle) and multiple lesions of low grade to a high degree of pancreatic intraepithelial neoplasia around the main lesion (black right triangle).

On admission, the patient did not have any complaints, and physical examination revealed no abnormalities. Laboratory test results showed WBC 8590/mm^3^, hemoglobin 12.2 g/dl, hematocrit 36.9%, platelet 287 × 10^9^/L, aspartate aminotransferase (AST) 31 U/L, alanine aminotransferase (ALT) 24 U/L, total bilirubin 0.5 mg/dl, alkaline phosphatase 65 IU/L, and CA 19–9 16 ng/ml (<37 ng/ml). Abdominal CT revealed a big 2.3 cm lesion at the remnant pancreas body (Figure 3). These findings suggested primary pancreatic cancer. There was no evidence of enlarged lymph nodes or peritoneal metastasis. The patient underwent curative completion total pancreatectomy comprising the remnant pancreas, spleen, and a jejunal segment including pancreaticojejunostomy. The operative time was 155 min, and the estimated blood loss was 120 ml. Histologic evaluation revealed moderately differentiated pancreatic ductal adenocarcinoma with perineural and lymphatic invasion; no lymph nodes were found positive for malignant cells; and there were multiple lesions of low grade to a high degree of pancreatic intraepithelial neoplasia (PanIN) around the main lesion, and the tumor stage was T2N0M0, stage IB, based on the TNM classification (Figure 4).

She was discharged on postoperative day (POD) 15. The patient has had no other complication for 13 months postoperatively and is currently keeping good health with adequate treatment with synthetic insulin and pancreatic enzyme supplements.

## Discussion

Bile duct cancer is a relatively rare cancer that occurs in the biliary tract system. From the standpoint of distal bile duct cancer, the overall 5-year survival of stages I, II, and III according to the stages based on the TNM stage was reported to be 59.0%, 35.4%, and 14.7%, respectively (6). Although chemotherapy and radiotherapy have been developed as treatment options, surgical resection provides the only chance of cure and long-term survival. However, long-term results after surgical resection of bile duct cancer remain unsatisfactory, and the survival rates are not good. However, the overall survival rate of distal bile duct cancer at 5 years was reported to increase to 27%–60% if R0 resection was achieved and from 0% to 40% when R0 resection was not performed (7, 8).

In a population-based study using the Surveillance, Epidemiology and End Results database between the years 1973 and 2015, the overall risk of developing second primary malignancy in patients with cholangiocarcinoma was significantly higher than that in the general population. Also, specific sites where the risk was significantly increased included transverse colon, intrahepatic bile duct, other biliary sites, and thyroid (9).

Within an English review of the literature in the MEDLINE, this is the first report about pancreatic ductal adenocarcinoma developed metachronously as second primary malignancy in a patient who underwent surgery for distal bile duct cancer. In this report, it is worth noting that the patient had survived 18 years without recurrence until pancreatic cancer was diagnosed, considering the bile duct cancer stage of T3N0M0, stage IIA. Although the patient underwent curative R0 resection, such long-term survival is not common after resection of distal bile duct cancer.

Moreover, if the survival of patients with bile duct cancer is prolonged, then second primary cancer may be encountered more commonly, as has been observed with other cancers. Be that as it may, the occurrence of both distal bile duct and pancreatic cancers in one patient is a rare phenomenon.

Fortunately, the patient received two curative surgical resections even 18 years apart for the two types of cancer that are considered as highly malignant tumors with aggressive biological behavior and poor prognosis. These two cancers apparently occurred independently, and each surgical treatment was successful. Therefore, we consider it important to follow up patients with bile duct cancer in order to check for second primary cancers, as well as recurrence, for long-term periods. Even for people with no signs of cancer remaining, the author recommends follow-up visits (that include CT scans and blood tests) approximately every 3 months for the first 3 years after surgery, and then approximately every 6 months for the next 6 years, and thereafter once a year. The importance of long-term follow-up in patients with pancreatobiliary cancer lies in early detection of recurrent or double primary cancer that can be cured with surgery, although there remains much to learn about identifying those at risk for the cancer.

In this study, genetic tests were not performed in the patient, and the etiology and pathogenesis of double primary cancers were consequently not discussed in depth. Despite this limitation, the results of our study are instructive. The early diagnosis of pancreatic cancer in the remnant pancreas should not be overlooked in patients treated for distal bile duct cancer, and the diagnosis of subsequent tumors must be taken into consideration during long-term follow-ups.

In conclusion, we observed the actual long-term recurrence-free survival of distal bile duct cancer in a patient who underwent PPPD and found that pancreatic cancer could occur in a remnant pancreas as secondary malignancy even with a time interval of 18 years, which was curatively treated by completion total pancreatectomy from early detection based on the long-term follow-up. This case highlights the need for long-term follow-up so that surgeons should be vigilant for the postoperative surveillance of patients with pancreatobiliary cancer in order to identify those in whom repeat surgical resection may be beneficial for this kind of metachronous cancer even after many years.

## Data Availability

The original contributions presented in the study are included in the article/Supplementary Material; further inquiries can be directed to the corresponding author.

## References

[1] Moertel CG Dockerty MB Baggenstoss AH. Multiple primary malignant neoplasms. I. Introduction and presentation of data. Cancer. (1961) 14:221–30. 10.1002/1097-0142(196103/04)14:2<221::AID-CNCR2820140202>3.0.CO;2-6 13771652

[2] Donin N Filson C Drakaki A Tan HJ Castillo A Kwan L Risk of second primary malignancies among cancer survivors in the United States, 1992 through 2008. Cancer. (2016) 122(19):3075–86. 10.1002/cncr.30164 27377470 PMC6192520

[3] Joo HJ Kim GH Jeon WJ Chae HB Park SM Youn SJ Metachronous bile duct cancer nine years after resection of gallbladder cancer. World J Gastroenterol. (2009) 15(27):3440–4. 10.3748/wjg.15.3440 19610150 PMC2712910

[4] Shima Y Okabayashi T Kozuki A Sumiyoshi T Tokumaru T Saisaka Y Completion pancreatectomy for recurrent pancreatic cancer in the remnant pancreas: Report of six cases and a review of the literature. Langenbecks Arch Surg. (2015) 400(8):973–8. 10.1007/s00423-015-1355-2 26545606

[5] Lahmar A Abid SB Arfa MN Bayar R Khalfallah MT Mzabi-Regaya S. Metachronous cancer of gallbladder and pancreas with pancreatobiliary maljunction. World J Gastrointest Surg. (2010) 2(4):143–6. 10.4240/wjgs.v2.i4.143 21160863 PMC2999228

[6] Jun SY Sung YN Lee JH Park KM Lee YJ Hong SM. Validation of the eighth American Joint Committee on cancer staging system for distal bile duct carcinoma. Cancer Res Treat. (2019) 51(1):98–111. 10.4143/crt.2017.595 29510611 PMC6333967

[7] Zhou Y Liu S Wu L Wan T. Survival after surgical resection of distal cholangiocarcinoma: a systematic review and meta-analysis of prognostic factors. Asian J Surg. (2017) 40(2):129–38. 10.1016/j.asjsur.2015.07.002 26337377

[8] Kwon HJ Kim SG Chun JM Lee WK Hwang YJ. Prognostic factors in patients with middle and distal bile duct cancers. World J Gastroenterol. (2014) 20(21):6658–65. 10.3748/wjg.v20.i21.6658 24914391 PMC4047355

[9] Zhuang L Yan X Meng Z. Second primary malignancy in patients with cholangiocarcinoma: A population-based study. Cancer Manag Res. (2019) 11:1969–83. 10.2147/CMAR.S187614 30881122 PMC6402443

